# Analysis of HIV-1 gp120 quasispecies suggests high prevalence of intra-subtype recombination

**DOI:** 10.1186/1742-4690-9-S2-P146

**Published:** 2012-09-13

**Authors:** IE Kiwelu, V Novitsky, L Margolin, J Baca, R Manongi, N Sam, J Shao, MF MacLane, SH Kapiga, M Essex

**Affiliations:** 1 Harvard School of Public Health, Boston, MA, USA; 2 Kilimanjaro Christian Medical College, Moshi, Tanzania, United Republic of; 3 Kilimanjaro Medical Christian College, Moshi, Tanzania, United Republic of; 4 London School of Hygiene and Tropical Medicine, London, UK

## Background

Recombination between viruses of the same HIV-1 subtype has been understudied primarily due to the lack of reference sequences, as well as appropriate bio-informatics tools. The introduction of recombination detection program (RDP3) made the detection of HIV-1 intra-subtype recombination feasible.

## Methods

This study determined the prevalence and patterns of HIV-1 intra-subtype recombinants among female bar and hotel workers in Moshi, Tanzania. The HIV-1 env gp120 V1-C5 quasispecies from 45 subjects classified as pure HIV-1 subtypes A1, C, or D were analyzed for recombination events by RDP3.

## Results

HIV-1 quasispecies with evidence for recombination were found in 89% of subjects infected with pure HIV-1 subtypes A, C, and D. Recombinant viruses were observed at both the baseline and the 12 month visits in 88% of the subjects; in 12% of subjects recombination was identified only at the later time point. Two major patterns were observed: 70% of subjects had unique recombination breakpoints without dominance of any particular variant, while in 30% of subjects a specific recombinant variant dominated in the pool of viral quasispecies.

## Conclusion

A large proportion of female bar and hotel workers were infected with intra-subtype recombinant viruses. These results suggest that HIV-1 co- and super-infections are common in this population, and occur more frequently than previously thought. Intra-subtype recombination contributes to the increased viral diversity which poses a challenge to HIV-1 vaccine design.

